# Loss of HSulf-1: The Missing Link between Autophagy and Lipid Droplets in Ovarian Cancer

**DOI:** 10.1038/srep41977

**Published:** 2017-02-07

**Authors:** Debarshi Roy, Susmita Mondal, Ashwani Khurana, Deok-Beom Jung, Robert Hoffmann, Xiaoping He, Eleftheria Kalogera, Thomas Dierks, Edward Hammond, Keith Dredge, Viji Shridhar

**Affiliations:** 1 Department of Experimental Pathology, Mayo Clinic, Rochester, MN, USA; 2 Division of Gynecologic Surgery, Mayo Clinic, Rochester, MN, USA; 3 Department of Chemistry, Biochemistry I, Bielefeld University, Bielefeld, Germany; 4 Zucero Therapeutics. Brisbane, Queensland, Australia

## Abstract

Defective autophagy and deranged metabolic pathways are common in cancer; pharmacologic targeting of these two pathways could provide a viable therapeutic option. However, how these pathways are regulated by limited availability of growth factors is still unknown. Our study shows that HSulf-1 (endosulfatase), a known tumor suppressor which attenuates heparin sulfate binding growth factor signaling, also regulates interplay between autophagy and lipogenesis. Silencing of HSulf-1 in OV202 and TOV2223 cells (ovarian cancer cell lines) resulted in increased lipid droplets (LDs), reduced autophagic vacuoles (AVs) and less LC3B puncta. In contrast, HSulf-1 proficient cells exhibit more AVs and reduced LDs. Increased LDs in HSulf-1 depleted cells was associated with increased ERK mediated cPLA2^S505^ phosphorylation. Conversely, HSulf-1 expression in SKOV3 cells reduced the number of LDs and increased the number of AVs compared to vector controls. Furthermore, pharmacological (AACOCF3) and ShRNA mediated downregulation of cPLA2 resulted in reduced LDs, and increased autophagy. Finally, *in vivo* experiment using OV202 Sh1 derived xenograft show that AACOCF3 treatment effectively attenuated tumor growth and LD biogenesis. Collectively, these results show a reciprocal regulation of autophagy and lipid biogenesis by HSulf-1 in ovarian cancer.

Previous reports have shown that downregulation of HSulf-1 is common in ovarian cancer (OvCa) and regulates heparan sulfate binding growth factor signaling which subsequently promotes tumorigenesis^1^. We recently reported that loss of HSulf-1 promotes a “lipogenic phenotype” as evidenced by an increase in lipid related metabolites, fatty acid synthesis and beta-oxidation, indicating an important role of HSulf-1 in metabolic regulation^2^.

Although adipocytes were described as the primary site for LD biogenesis^3^,^4^, recent findings suggest that lipid droplets (LDs) may be an important source of energy in cancer cells^5^,^6^,^7^. Enhanced LD biogenesis in cancer cells plays a sentinel role in cell signaling, membrane trafficking and lipid metabolism, all associated with increased growth and survival of cancer cells^8^,^9^. LDs are considered cellular hallmarks of many different diseases such as diabetes, atherosclerosis and cancer^8^,^10^,^11^,^12^,^13^. Recent findings have shown higher LD amount in colon cancer stem cell population compared to their differentiated counterparts indicating more important function of LDs in cancer progression^14^. Cancer cells rich in LDs are also shown as chemoresistant in nature which further suggests the critical role of LDs in survival of cancer cells^15^.

Although the presence of LDs is associated with disease progression, the functional significance in promoting inflammation and tumorigenesis is not well understood. More importantly, the molecular alterations that promote LD accumulation in cancer cells have not been described.

Primarily, LDs are storage organelles for neutral lipids and cholesterol esters^16^. Stress-induced release of fatty acids from the stored LDs provides energy which subsequently promotes tumor growth, metastasis and cell survival of OvCa^17^. Several of the LD associated proteins involved in LD biogenesis and release of fatty acids, such as *SREBP1, PLINs, PLA2G4A, PLA2G3, ATGL, HSL, MAGL, PPARγ*, are upregulated upon HSulf-1 loss^3^,^18^,^19^,^20^,^21^,^22^,^23^. Many of these genes are overexpressed in cancer cells and have been implicated as potential contributors towards tumorigenesis^3^,^24^,^25^,^26^,^27^.

Autophagy is a lysosomal pathway by which long-lived proteins and damaged organelles are degraded to provide energy to the cell and maintain cellular homeostasis^28^,^29^,^30^. Although it is generally accepted that LDs are substrates for lipases, Singh *et al*.^31^,^32^. identified LD as substrates for macro-autophagy and coined the term lipophagy to describe the engulfment of LDs by autophagosomes which ultimately fuse with lysosomes for the breakdown of LD components under stress conditions. Following this seminal finding, there have been several reports on the regulation of LDs by autophagy^33^,^34^,^35^. However, the interplay between the molecular components of these two metabolic pathways is not known in OvCa. In the present work, we identify HSulf-1, a known major regulator of growth factor signaling, as the missing link between autophagy and LDs in OvCa acting through cPLA2α in order to promote LDs and inhibit autophagy.

## Results

### HSulf-1 loss promotes lipid droplet biogenesis and defective autophagy

**Figure 1:**
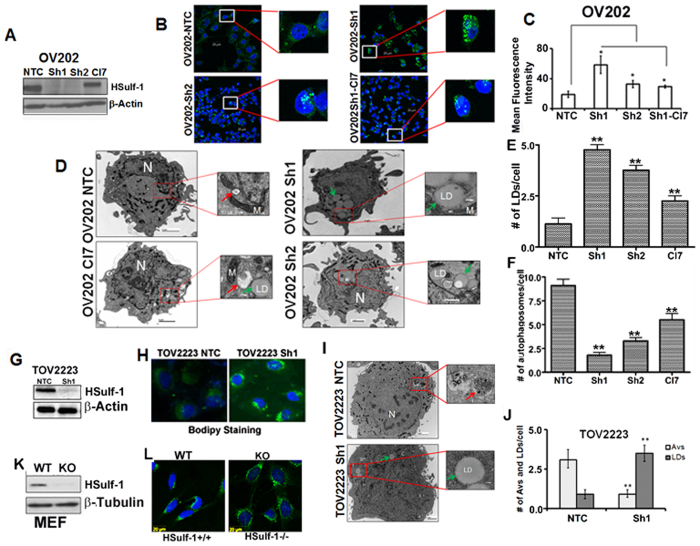
HSulf-1 loss promotes lipid droplet biogenesis and defective autophagy in OV202, TOV2223 and HSulf-1 knockout mouse embryonic fibroblast cells. (**A**) Protein expression of HSulf-1 was assessed by western blot in OV202 clones (NTC, Sh1, Sh2 and Cl7 cells). β-Actin was probed as a loading control. (**B**) OV202NTC, Sh1, Sh2 and Cl7 cells were labeled with Bodipy 493/503 (green) and DAPI (Blue) to determine the cytoplasmic lipid droplets (LD) and nuclei respectively. (**C**) Mean fluorescence of Bodipy in NTC, Sh1, Sh2 and Cl7 is measured using Carl Zeiss Zen (2009) software and the data is plotted as a bar diagram. Sh1 and Sh2 cells were compared to NTC cells whereas Cl7 cells were compared to Sh1 cells. In both the cases, (p ≤ 0.05) (**D**) Representative trans-electron microscopic (TEM) images of NTC, Sh1, Sh2 and Cl7 cells are shown; cytoplasmic LDs are marked with green arrows, whereas red arrows indicate autophagic vesicles (AV). (**E** and **F**) LDs and AVs quantified in NTC, Sh1 and Sh2 cells and plotted in a bar graph. Sh1 and Sh2 cells were compared to NTC cells and, Cl7 cells were compared to Sh1 cells. In all the cases p ≤ .0.01 (**G**) Protein expression of HSulf-1 in TOV2223 NTC and Sh1 cells is detected by immunoblot analysis; β-actin was probed as a loading control. (**H**) Bodipy staining performed in TOV2223 NTC and Sh1 cells to detect LDs. (**I**). Representative TEM images of TOV2223 NTC and Sh1 cells are shown. (**J**) AVs and LDs in TOV2223 cells are quantified. We compared TOV2223 NTC cells to TOV2223 HSulf-1 knockdown Sh cells and found significant differences in the # of AVs and LDs (**K**) Protein expression of HSulf-1 in Mouse Embryonic Fibroblast (MEF) wild type (WT) and HSulf-1 knock out (Sulf-1^−/−^) cells is detected by western blotting. β-Tubulin was used as a loading control. (**L**) MEF cells were labeled with Bodipy 493/503 (green) to determine cytoplasmic LDs. Data are shown as mean ± SD of 3 replicates per treatment. *P < 0.05; **P < 0.01.

We previously reported that stable genetic downregulation of HSulf-1 in OV202 cells, achieved by two different shRNAs targeting HSulf-1 (OV202Sh1 and Sh2 cells), exhibited increased LDs compared to non-targeted control transduced cells (NTC)^2^. We further confirmed these findings using OV202Sh1 to OV202Sh1 clone Cl7 cells (generated by re-expression of CMV-driven HSulf-1 construct in Sh1 cells). HSulf-1 levels were confirmed in these clones by western blot analysis as shown in Fig. 1A. Subsequently, LDs were detected with Bodipy staining in these cells. Rescue of HSulf-1 in OV202Cl7 cells reduced the number of LDs compared to OV202Sh1 and Sh2 cells (Fig. 1B, insets). Mean fluorescence intensity of Bodipy staining in these cells is shown in Fig. 1C.

We further performed quantification of the changes in the LDs by Transmission Electron Microscopy (TEM). Data showed significantly higher numbers of LDs in OV202Sh1 and Sh2 cells compared to NTC cells (Fig. 1D and E). The increased LDs indicate the nutrient rich or energy proficient state of the cells and, therefore, we hypothesized that cells will not activate catabolic process such as autophagy under these conditions. In order to verify, we measured the extent of autophagy via TEM analysis in these cells and observed that HSulf-1-depleted OV202Sh1 and Sh2 cells exhibited less autophagic vesicles (AVs) when compared to OV202NTC cells (Fig. 1F). Also, rescue of HSulf-1 in OV202Cl7 cells resulted in increased AV compared to OV202Sh1 cells. Higher resolution TEM images of OV202 cells are provided in Fig. S1. To rule out the possibility of cell-specific effect, downregulation of HSulf-1 in TOV2223 cells with ShRNA (Fig. 1G) also showed increased numbers of LDs in TOV2223Sh1 cells compared to TOV2223NTC cells both by Bodipy staining (Fig. 1H) and TEM analysis (Fig. 1I). These results indicate that these metabolic alterations are not unique to the OV202 cell line alone. Similarly, a higher number of AVs was observed in both OV202NTC and TOV2223NTC cells as compared to the respective HSulf-1-depleted cells. The quantification of LDs and AVs in TOV2223 cells is shown in Fig. 1J. In addition, HSulf-1 knockout (KO) mouse embryonic fibroblasts (MEFs) also displayed increased numbers of LDs compared to wild-type MEFs (Fig. 1L) with HSulf-1 expression (Fig. 1K). Similarly, ectopic expression of HSulf-1 in SKOV3 cells resulted in a significant decrease in the numbers of LDs compared to vector-transfected controls (Fig. S2B). Vector-transfected SKOV3 cells exhibited lesser degree of AVs as quantified through TEM analysis, compared to HSulf-1 transfected SKOV3 cells (Fig. S2C). Quantification of LDs and AVs in 25 cells is shown in Fig. S2D.

### Cytosolic phospholipase A2 (cPLA2) is activated/phosphorylated in HSulf-1 depleted ovarian cancer cells

**Figure 2:**
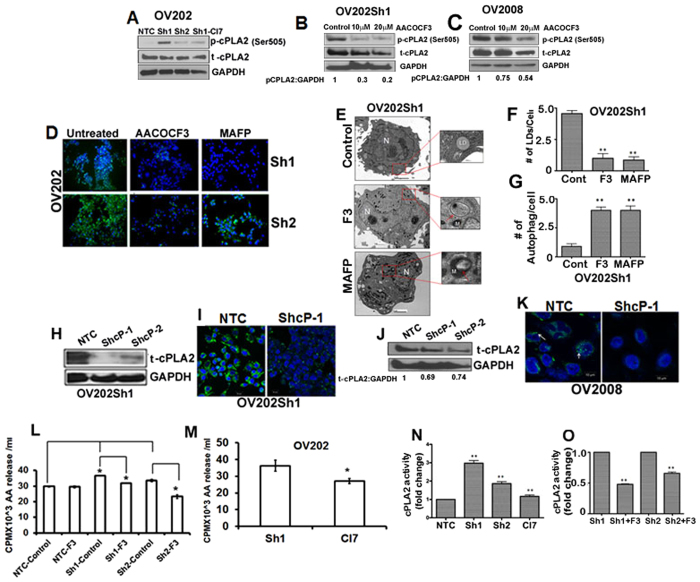
Inhibition of cPLA2 attenuates lipid droplet biogenesis in OV202Sh1 cells. (**A**) Immunoblot analysis shows p-cPLA2 and t-cPLA2 levels in OV202 NTC, Sh1, Sh2 and Cl7 cells. (**B** and **C**) Immunoblot analysis shows p-cPLA2 and total-cPLA2 levels in OV202 Sh1 and OV2008 cells after treatment with 10 μM and 20 μM of cPLA2 inhibitor and AACOCF3, respectively. (**D**) Sh1 and Sh2 cells were treated with either 10μM of AACOCF3 or 10μM of MAFP for 24 hours followed by Bodipy (493/503) staining to identify lipid droplets (LD). (**E**) Representative trans-electron microscopic (TEM) images of OV202 Sh1 cells untreated and treated with 10 μM of AACOCF3 (F3) and 10 μM of MAFP for 24 hours. LDs are marked with green arrows, whereas red arrows indicate autophagic vesicles (AV). (**F** and **G**) Quantification of LDs and AVs from the TEM images presented in a bar graph.(**H** and **J**). Total cPLA2 expression was transiently downregulated with two different ShRNAs targeting the open reading frame of cPLA2 in OV202Sh1 and OV2008 cells. Immunoblot analysis shows total-cPLA2 expression in NTC, ShcP-1 and ShcP-2 cells. (**I** and **K**) Bodipy staining of LDs in OV202 Sh1 and OV2008 cells after transiently transfecting them with either empty vector or Sh-cPLA2. (**L**) Arachidonic acid (AA) release is evaluated in OV202 NTC, Sh1, and Sh2 cells treated or untreated with 10 μM of AACOCF3 (F3). Cells were incubated with H^3^-AA under serum starved condition for 24 hours. Fresh medium was added to the cells after washing and aliquots of growth medium were measured for radioactivity after 24 hours. Radioactivity is presented as counts per minute (cpm)/ml. (**M**) AA release was evaluated in OV202 Sh1 and Cl7 cells. (**N**) cPLA2 enzyme activity was measured in OV202 NTC, Sh1, Sh2 and Cl7 cells using the cPLA2 activity assay kit. (**O**) cPLA2 activity was also determined in OV202 Sh1 and Sh2 cells after treating them with AACOCF3. AA release and cPLA2 activity assay experiments were repeated three times. Data are shown as mean ± SD of 3 replicates per treatment. *P < 0.05; **P < 0.01. Band intensity was quantified using Image J software.

We next wanted to determine the underlying mechanism by which HSulf-1 depletion leads to increased LD accumulation in OvCa cells. LDs accumulate following activation of an anabolic process known as lipid biosynthesis. We showed earlier that LD-associated proteins were upregulated in HSulf-1 depleted cells. Among them, cytosolic phospholipase A2 _α_ (cPLA2α) has been previously proven to be a key protein in LD biogenesis^36^. Specifically, activation of cPLA2α by phosphorylation at Ser505 by p-ERK has been shown to be a critical step in the cPLA2-mediated LD biogenesis^37^. Thus, we first sought to determine the activated/phosphorylated levels of cPLA2α by Immunoblot analysis. Our data show that p-cPLA2^ser505^ was clearly increased in OV202Sh1 and, to a lesser extent, in OV202Sh2 cells (Fig. 2A). Conversely, re-expression of HSulf-1 reduced the cPLA2 phosphorylation, indicating that increasing levels of HSulf-1 reversed phosphorylation. Similarly, we found higher p-cPLA2 levels in TOV2223 HSulf-1 knockout cells compared to the TOV2223 NTC cells (Fig. S3) Quantification of p-cPLA2 (Fig. 2B and C) and t-cPLA2 (Fig. 2J) is shown below their respective immunoblots.

### Pharmacological Inhibition of cPLA2 attenuates lipid droplet biogenesis in OV202Sh1 cells

To further investigate the role of cPLA2 activation and LD accumulation/biosynthesis, we included an additional cell line, OV2008 deficient in HSulf-1 expression that expressed both high amounts of p-cPLA2 and LDs. We treated OV202Sh1 and OV2008 cells with cPLA2 specific inhibitors, AACOCF3 and MAFP (10 and 20 μM). The selected doses of cPLA2 inhibitors showed no toxicity to the cells as determined by LDH release assay (Fig. S9). Western blot analysis show decreased levels of p-cPLA2 in inhibitor-treated cells (Fig. 2B and C). Bodipy staining upon inhibition of cPLA2 activity with 10 μM of AACOCF3 and MAFP in OV202Sh1 and OV202Sh2 cells showed almost complete inhibition of LD biogenesis in these cells compared to untreated control cells (Fig. 2D). Consistent with this data, TEM analysis of OV202Sh1 cells with AACCOF3 and MAFP also revealed significantly lower number of LDs and higher number of AVs (Fig. 2E). Quantitation of LDs and AVs in 25 cells is shown in Fig. 2F and G. Furthermore, transient downregulation of cPLA2 expression with two different ShRNAs (ShcP-1 and ShcP-2) against cPLA2 in OV202Sh1 cells (Fig. 2H) resulted in a decrease in the number of LDs as shown by Bodipy staining (Fig. 2I). Similarly, stable downregulation of cPLA2 in OV2008 cells (Fig. 2J) resulted in reduced numbers of LDs (Fig. 2K). To determine whether cPLA2 inhibitors were able to inhibit cPLA2 activity, we performed arachidonic acid (AA) release assay in OV202Sh1 and OV202Sh2 cells. Cells treated with 10 μM of AACOCF3 showed significant reduction of AA release indicating that activity of cPLA2 was inhibited (Fig. 2L and M). Further, cPLA2 activity in these cells was measured using cPLA2 activity assay kit (Fig. 2N and O). cPLA2 activity in OV202 Sh1 and Sh2 cells was almost 3-fold and 2-fold higher compared to NTC cells. OV202Cl7 cells showed reduction in cPLA2 activity compared to Sh1 cells. cPLA2 activity was downregulated in Sh1 and Sh2 cells when treated with AACOCF3 (Fig. 2O).

### Inhibition of growth factor-mediated signaling reduced p-cPLA2 and lipid droplet biogenesis

**Figure 3:**
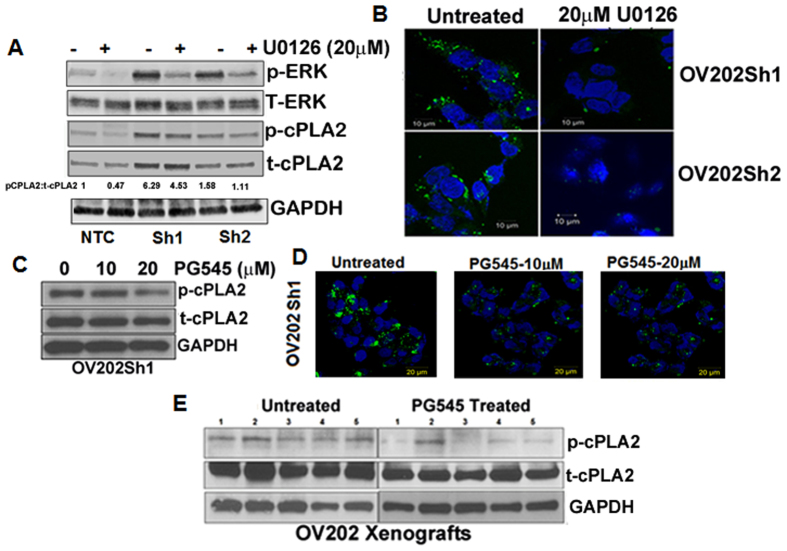
Inhibition of growth factor mediated signaling reduced p-cPLA2 and lipid droplet biogenesis. (**A**) OV202 NTC, Sh1 and Sh2 cells were treated for 24 hours with 20μm of U0126, a MEK inhibitor, followed by western blot analysis to detect the protein expression of p-ERK, t-ERK, p-cPLA2, t-cPLA2 and GAPDH. Band intensity was quantified using Image J software. (**B**) Bodipy staining for lipid droplets (LD) in OV202 Sh1 and Sh2 cells after treatment with 20μM U0126. (**C**) Immunoblot analysis of p-cPLA2 and t-cPLA2 in OV202 Sh1 cells is shown after treatment with 10 and 20μM PG545. (**D**) Bodipy staining of LDs in OV202 Sh1 cells after treating them with 10 and 20μM of PG545. (**E**) Immunoblot analysis of p-cPLA2 and t-cPLA2 in lysates from untreated and PG545 treated OV202 Sh1 xenografts. Data shown in Fig. 3(**A** to **D**) is a representative image from 3 independent experiments.

cPLA2 is activated by phosphorylation at serine residues 505, 515, and 727^38^. Within these sites, Ser-505 phosphorylation leads to increased catalytic activity^39^, which depends on MAPK, ERK and p38^39^,^40^,^41^. We previously showed that downregulation of HSulf-1 in OV202 cells results in increased ERK activity^42^. Conversely, re-expression of HSulf-1 in HSulf-1 deficient cells results in attenuation of growth factor-mediated ERK activity^43^,^44^,^45^. To determine if inhibition of ERK activity will alter p-cPLA2 levels, OV202NTC, OV202Sh1 and OV202Sh2 cells were treated with 20 μM U0126 (MEK inhibitor) for 24 hours. As shown in Fig. 3A, treatment with U0126 resulted in the attenuation of p-ERK and p-cPLA2^ser505^ levels in OV202Sh1 and OV202Sh2 cells compared to NTC cells. Bodipy staining of OV202Sh1 and OV202Sh2 cells shows reduced LD accumulation as a result of U0126-mediated inactivation of ERK and p-cPLA2 (Fig. 3B). Bodipy staining in Sh1 and Sh2 cells were quantified using Imagej software (Fig. S4)

To further elucidate the role of growth factor signaling, we treated OV202Sh1 cells with PG545, a clinically-relevant heparan sulfate (HS) mimetic that mimics the action of HSulf-1^46^, and evaluated the expression levels of total and p-cPLA2 (Fig. 3C). PG545 blocks HS-mediated growth factor signaling, similar to HSulf-1, inhibits angiogenesis and carcinogenesis^47^ in various cancers including OvCa^48^. Our recent report indicated that PG545 effectively inhibited OV202Sh1-induced tumorigenesis and also modulated levels of glycolytic enzymes both *in vitro* and *in vivo*^46^. In the current study, we demonstrated for the first time that PG545 treatment (20 μM) reduced the level of p-cPLA2, suggesting a role in the regulation of lipid metabolism. Consistent with this finding, PG545 treatment reduced the number of LDs in OV202Sh1 cells (Fig. 3D). Bodipy staining was further quantified using Image J software (Fig. S4B). No toxicity was observed in OV202Sh1 cells from the PG545 dosage used in our study as determined by LDH release assay (Fig. S9)

To evaluate the effect of PG545 on lipid metabolism *in vivo*, we determined the level of p-cPLA2 in PG545-treated OV202Sh1 xenografts^46^ by immunoblot analysis. As shown in Fig. 3E, PG545 reduced the levels of p-cPLA2 in the treated xenografts compared to untreated controls. Collectively, these results indicate that extracellular growth factor signaling is directly associated with LD biogenesis.

### Defective autophagy is associated with loss of HSulf-1

**Figure 4:**
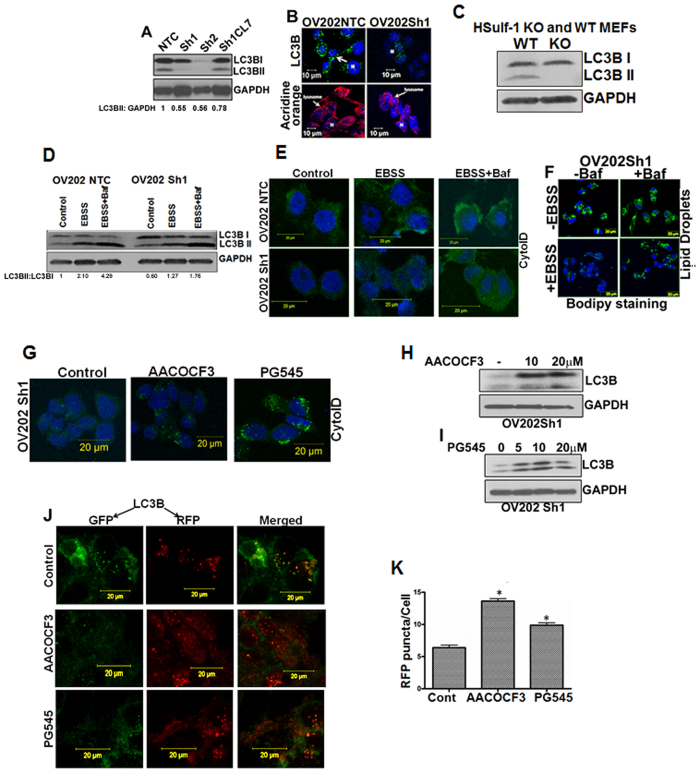
Dysfunctional autophagy is associated with loss of HSulf-1 and activation of cPLA2. (**A**) Immunoblot analysis with anti-LC3B antibody in OV202 NTC, Sh1, Sh2 and Cl7 cells. (**B**) Immunoflourescence analysis of OV202 NTC and Sh1 cells with anti-LC3B antibody (green, top panels), acridine orange (red, lower panels) and DAPI (blue). (**C**) Immunoblot analysis of LC3BI and LC3BII in HSulf-1 knockout Mouse Embryonic Fibroblast (MEF) and wild type cells were performed. GAPDH was used as a loading control. (**D**) Immunoblot analysis of LC3Bin OV202 NTC and Sh1 cells treated with EBSS with or without bafilomycin A1 (100 nM) for 2 hours. GAPDH was used as a loading control. LC3BII to GAPDH ratio is also presented in this figure. (**E**) Cyto-ID staining of OV202 NTC and Sh1 cells in control, EBSS and EBSS + Baf treated cells. Cells were grown in either regular media or EBSS to induce autophagy. (**F**) Bodipy staining of lipid droplets (LD) in OV202 Sh1 cells treated with EBSS with or without bafilomycin A1 (100 nM) for 1 hour. Cells grown in complete medium were used as controls (without EBSS). (**G**) Immunofluorescence analysis of Cyto-ID staining of OV202 Sh1 cells treated with 20 μM of AACOCF3 and 20 μM of PG545 showing increase in autophagy. (**H** and **I**) Immunoblot analysis of LC3BII levels in OV202 Sh1 cells treated with increasing concentrations of AACOCF3 and PG545, respectively. (**J**) Following transient expression of Cherry-GFP-LC3B (48 hours of incubation), autophagic flux was assessed in OV202 Sh1 cells treated with AACOCF3 and PG545 for 24 hours. Confocal microscopy analysis showed changes in the autolysosome formation. Representative images from two independent experiments are shown. (**K**) Quantification of RFP puncta is presented in a bar diagram. 25 cells were quantified for fluorescence measurement. Data are shown as mean ± SD for 3 replicates per treatment. *P < 0.05.

Previous reports suggest that enhanced lipid metabolism may lead to autophagic impairment^35^,^49^. To understand if HSulf-1 loss mediated lipogenic phenotype of OV202Sh1/Sh2 cells is associated with an altered autophagic response, we analyzed the lipidated form of LC3BII levels as one of the ways to evaluate autophagy^50^,^51^ by immunoblot analysis. As shown in Fig. 4A, while the HSulf-1 proficient OV202NTC and Cl7 cells (rescue clone) expressed LC3BII, the HSulf-1 deficient OV202Sh1 and OV202Sh2 cells had negligible levels of LC3BII. Consistent with these findings, less LC3B puncta and acidic vesicles were observed in OV202Sh1 cells compared to NTC cells using immunofluorescence (IFC) analysis and acridine orange staining (Fig. 4B). To further confirm the role of HSulf-1 in autophagic response, immunoblot analysis of LC3B was performed in wild-type (WT) MEFs and HSulf-1 KO MEFs. HSulf-1 KO MEFs expressed only LC3BI and no LC3BII, whereas the WT MEFs expressed both LC3BI and LC3BII (Fig. 4C).

Differences in autophagic flux in OV202 NTC and Sh1 cells were determined by staining the cells with an autophagy specific dye, Cyto-ID. Cells were grown in EBSS medium (amino acid deleted medium) for 2 hours to induce autophagy as well as in the presence of 100 nm of bafilomycin to inhibit autophagic degradation in lysosomes. Exposure to EBSS-induced autophagy resulted in higher levels of autophagy in the OV202NTC cells compared to the OV202Sh1 cells as evidenced by increased Cyto-ID staining (used to detect autophagosomes and autolysosomes) in the OV202NTC cells (Fig. 4D, middle panel, top row); this result was even more evident upon bafilomycin treatment (Fig. 4D, panel 3). Exposure to EBSS-induced autophagy resulted in higher levels of autophagy in the OV202NTC cells compared to the OV202Sh1 cells as evidenced by increased Cyto-ID staining (used to detect autophagosomes with minimal staining of the lysosomes) in the OV202NTC cells (Fig. 4E, middle panel, top row); this result was even more pronounced upon bafilomycin treatment (Fig. 4E, panel 3). Bafilomycin treatment inhibits fusion of autophagosomes to lysosomes and hence accumulates more autophagosomes in the cell resulting in an increased cyto-ID staining.

### Autophagy induction abrogates lipid droplet biogenesis in OV202Sh1 cells

Previous reports demonstrated that inhibition of AV in rat hepatocytes resulted in increased accumulation of triglycerides in the form of LDs and that activation of autophagy decreased LD levels^32^. Based on these studies, we next examined the consequences of inducing autophagy on LDs in OV202Sh1 cells. EBSS treatment of OV202Sh1 cells resulted in reduction of LDs accumulation (Fig. 4F, lower left panel). Furthermore, inhibition of autophagy by bafilomycin rescued LDs; this suggests that autophagy-mediated lipophagy may be responsible for the reduction in the level of LDs in HSulf-1 deficient cells (Fig. 4F, lower right panel). Quantification of Cyto-ID and Bodipy staining is determined using Image J software and represented as CTCF Fig. S5A and B.

Our data as shown in Fig. 2D, consistent with previous reports by other groups^36^,^52^, indicated that inhibition of p-cPLA2 activity with AACOCF3 abrogated LD biogenesis in OV202Sh1 cells. Based on the concept that lipid accumulation beyond a certain physiological level impairs autophagic function^31^, we hypothesized that AACOCF3 inhibition of p-cPLA2 activity, resulting in inhibition of LDs, may promote autophagy. Treatment of OV202Sh1 cells with AACOCF3 revealed induction of autophagy as shown by increased Cyto-ID staining with IFC (Fig. 4G) and increased LC3BII levels with immunoblot analysis (Fig. 4H, top panel). Taking into consideration prior studies that proved that increased growth factor signaling is associated with increased lipogenesis^53^,^54^,^55^,^56^, in conjunction with our earlier data showing that PG545 treatment decreases the number of LDs in OV202Sh1 cells (Fig. 3D), we next determined the level of autophagy following PG545 treatment of OV202Sh1 cells using Cyto-ID staining. We observed an increase in the Cyto-ID staining following PG545 treatment (Fig. 4G**, right panel**) as well as an increase in LC3BII levels by immunoblot analysis (Fig. 4I). Notably, similar to OV202NTC and OV202Sh1 cells, HSulf-1 KO MEFs showed negligible levels of autophagy compared to WT MEFs (Fig. S6A**, upper panel**). More importantly, EBSS treatment induced autophagy to a greater extent in WT MEFs compared to HSulf-1 KO MEFs as evidenced by Cyto-ID staining; this effect was even more pronounced following bafilomycin treatment (Fig. S6A, middle and right upper panels respectively). In addition, AACOCF3 and PG545 treatment of HSulf-1 KO MEFs resulted in induction of autophagy and reduction of LDs (Fig. S6B). These data indicate that diminished autophagic flux in HSulf-1 deficient cells is associated with increased accumulation of LDs.

Lipid accumulation beyond a certain physiological level impairs autophagic function, as previously discussed. In agreement with this, addition of exogenous lipids in the form of BSA conjugated palmitate in OV202NTC enhanced LD accumulation in the cytoplasm and inhibited autophagy (Fig. S7, top and lower panel, respectively). Additionally, there was greater LD accumulation in the ATG5 KO MEFs cells compared to the WT MEFs. In contrast, ATG5 KO cells exhibited a lower level of Cyto-ID staining compared to the WT cells (Fig. S8). These results provide evidence to suggest that there is a relationship between induction of autophagy and abrogation of LDs.

Although increase in LC3BII is indicative of autophagic flux, a more reliable assay to monitor autophagic flux is the use of fluorescent-tagged Cherry-GFP-LC3B construct^55^,^56^,^57^,^58^ following autophagy induction^52^,^53^. As the GFP signal is pH sensitive and quenched in the lysosomes, autophagosomes light up as both red and green, while autolysosomes light up as mostly red (seen as orange to yellow in merged images). Following transient transfection for 48 hours of OV202Sh1 cells with Cherry-GFP-LC3B, cells were treated with either 20 μM of AACOCF3 or 20 μM of PG545. Induction of autophagy by these agents resulted in an increase in Cherry positive signals in the treated cells (Fig. 4J, panels 2 and 3, respectively) compared to untreated control cells (Fig. 4J, panel 1); this is clearly indicative of activation of autophagic flux. Quantification of red fluorescent protein (RFP) puncta/cell is provided in Fig. 4K.

### AACOCF3 alone and in combination with carboplatin suppresses tumor growth and inhibits lipid droplet biogenesis *in vivo*

In order to test whether there is an added benefit in combining AACOCF3 with carboplatin (CBP), which is a platinum-based drug that constitutes standard of care for ovarian cancer, we used the Chou Talalay methodology to test a synergistic effect when combined *in vitro as described in the methods section*. Interestingly, when the two drugs were combined in an equipotent combination (IC_50_:IC_50_ ratio), Sh1 cells exhibited synergistic effect (combination index <0.9) at the clinically relevant range of fraction affected (Fa; Fa>0.5) (Fig. S10). This supported our hypothesis that combination of these drugs *in vivo* may lead to a more pronounced effect than each drug alone.

**Figure 5:**
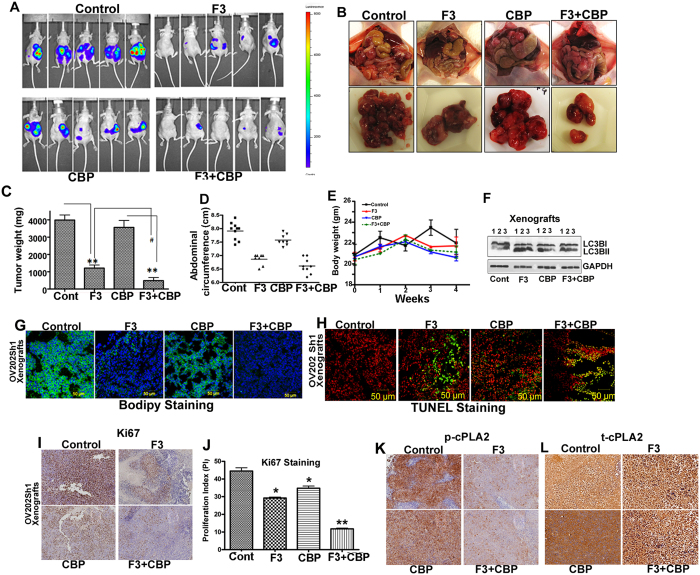
AACOCF3 alone and in combination with carboplatin suppresses tumor growth, and inhibits lipid droplet biogenesis *in vivo.* (**A**) Randomized OV202 Sh1 tumor-bearing mice (10 mice per group), were treated with water, AACOCF3 (F3) (10 mg/kg), carboplatin (CBP) (51 mg/kg) or with a combination of AACOCF3 and CBP for 4 weeks as detailed in the manuscript. Mice were euthanized after 28 days. Representative images of the mice prior to sacrifice from control, F3, CBP, and CBP + F3 treatment groups using IVIS luminescence imaging system series 2000 are presented. Color barn indicates photon intensity. (**B**) Representative images of excised tumors from OV202 Sh1 xenografts. (**C**) Excised tumor weights from control, F3, CBP, and CBP + F3 treatment groups. (**D**) Abdominal circumference of the mice measured on the day of sacrifice across treatment groups. (**E**) Total body weight of control, F3, CBP, and CBP + F3 treatment groups.(**F**) Immunoblot analysis of LC3BI and LC3BII in lysates from control, F3, CBP, and F3 + CBP treatment groups. GAPDH was used as loading control. (**G**) Tumor xenografts were stained with Bodipy stain to detect lipid droplets (LD). DAPI was used to stain the nuclei. (**H**) Representative images of TUNEL staining of frozen section of xenografts from control, F3, CBP, and CBP + F3 treatment groups. Green fluorescence indicates TUNEL and red fluorescence indicates propidium iodide staining. (**I**) Representative images of immunohistochemical staining of Ki67. (**J**) Bar graph showing the Ki67 proliferation index (*P < 0.05 and **P < 0.01). The Ki67 proliferation index was measured using ImmunoRatio (public domain image analysis software). (**K**) p-cPLA2 in formalin-fixed, paraffin-embedded sections of treated and untreated xenografts.

The effect of AACOCF3 alone and in combination with CBP on primary tumor growth was evaluated in OV202Sh1 cells bearing nude mice. A total of 5 × 10^6^ cells (in serum-free RPMI 1640), from Sh clones expressing luciferase, were injected intraperitoneally into female athymic nu/nu mice at 4 to 5 weeks of age (National Cancer Institute, Frederick Animal Production Area, Frederick, MD). Once intraperitoneal implants were visible via non-invasive imaging (approximately 4 days after inoculation), mice were randomized into groups (10 mice/group) and treated with intraperitoneal injection of 10 mg/kg of cPLA2 inhibitor, AACOCF3 (referred to as F3 in the figures), every third day until the end of the study, 51 mg/kg of CBP every 5 days until the end of the study, and a combination of CBP + F3 every 5 days, as described in the methods. Luciferase imaging of representative mice from all four groups (vehicle control and 3 treatment groups) is shown in Fig. 5A. Higher luciferase intensity in the control and CBP groups indicates increased tumor volume, progression, and metastasis. Image of representative tumor specimen from each group at time of necropsy is shown in Fig. 5B. Comparison of the mean abdominal circumference and tumor weight of the mice across groups at time of necropsy revealed that combination treatment was more effective in halting cancer progression compared to all other groups (Fig. 5C and D). There was no significant body weight loss in F3, CBP, or combination treatment groups compared to control group suggesting that F3, CBP as well as combination treatment were well tolerated without apparent toxicity to the mice (Fig. 5E). Western blot analysis of lysates from F3 alone and F3 + CBP combination treated xenografts showed an enhanced LC3B-II level compared to the untreated control and CBP alone xenografts as shown in Fig. 5F. Importantly, Bodipy staining frozen sections of xenograft showed significantly higher levels of LDs in the control and CBP groups compared to the F3 and combination groups (Fig. 5G); this is consistent with the *in vitro* data shown in Fig. 2D, top panel. In contrast, more intense TUNEL staining was observed in the F3 and combination groups compared to control and CBP groups (Fig. 5H). Immunohistochemistry analysis of AACOCF3 monotherapy and combination treatment with CBP correlated with significant reductions in the levels of tumor cell proliferation markers Ki67, p-cPLA2 and t-cPLA2 (Fig. 5I, K and L).

## Discussion

LD biogenesis is a mechanism by which cells store excess free fatty acids in the form of triacylglycerides and cholesteryl esters^59^. Under stress conditions, free fatty acids are released by lipolysis from the LDs and undergo beta oxidation in the mitochondria to provide energy to the dying cell^3^. During oncogenic transformation, many cancer cells manifest a high rate of *de novo* lipid synthesis which, in turn, results in accumulation of cytoplasmic LDs^43^,^53^,^60^. We recently reported that loss of HSulf-1 promotes a “lipogenic phenotype” by upregulating fatty acid synthesis and beta-oxidation leading to accumulation of lipid-associated metabolites; this finding indicates an important role of HSulf-1 in cancer-related metabolic regulation^2^. In this study, we identified HSulf-1 as a molecular link between LD accumulation and autophagy.

Autophagy maintains the cellular homeostasis by recycling cellular components such as lipids and proteins^29^. Previous studies have shown that increased growth factor signaling, (which is predominant in HSulf-1 deficient cells) may lead to reduced autophagy^61^. In contrast, limited growth promoting signaling and nutrient depletion are two known positive regulators of autophagy. In this study, we show that HSulf-1 expressing cells have attenuated ERK-1/2 activation, resulting in diminished cPLA2 activation, a key protein involved in lipid biogenesis. Similarly, loss of HSulf-1 results in marked decrease in autophagic flux and significant increase in LDs accumulation. This is particularly relevant as it is well recognized that autophagic clearance of LDs maintains the cellular lipid homeostasis in a steady state, and that, conversely, impaired autophagy results in accumulation of lipids in the form of LDs leading to tumor growth and cancer progression^31^,^53^,^62^,^51^,^60^. A recent report by Xu *et al*. has estabslihed a close association between lipid droplet clearence by autophagic degradation mechanism to the survival of renal cancer cell carcinoma patients^51^. Taking these findings into consideration, we hypothesize that loss of HSulf-1, may play a role in promoting a lipogenic phenotype by increasing LD biogenesis as well as interfering with autophagic clearance of cytoplasmic LDs. A recent report by Ishihara *et al*. has clearly indicated the role of cPLA2 in the regulation of autophagy which further supports our study^63^. Consistent with this hypothesis, we have previously reported that loss of HSulf-1 also promotes enhanced fatty acid oxidation in Sh1 and Sh2 cells compared to NTC cells. Additionally, we demonstrated that supplementation of BSA conjugated palmitic acid in the OV202 NTC cells promoted cytoplasmic LD accumulation while inhibiting autophagy, lending further to support the existence of an inverse relation between LD biogenesis and autophagy.

This is the first report to link loss of HSulf-1 with increased cPLA2 activity which in turn triggers LD biogenesis. Consistent with LD accumulation, OV202Sh cells showed an increased AA release compared to NTC cells. Released AA is critical for inducing inflammatory responses in cancer cells though cyclooxygenase and lipoxygenase pathways^64^. Pharmacological inhibition of cPLA2 phosphorylation reduced AA release and LD biogenesis, suggesting that loss of HSulf-1 mediated LD biogenesis and AA release is dependent on cPLA2 activity.

We recently reported that OV202 Sh1 cells treated with PG545, a HS mimetic that mimics the action of HSulf-1 in inhibiting HS binding growth factor signaling^46^, resulted in significant reduction in tumor growth^46^. In this study, we show that PG545 diminished p-cPLA2 levels, decreased the number of LDs and enhanced autophagy in OV202Sh1 cells. Importantly, EBSS treatment promoted autophagy and reduction in LDs in HSulf-1 KO MEFs supporting the role of HSulf-1 in regulating these two interconnected metabolic pathways *in vivo*. In contrast to a recent report by Shteingauz *et al*.^65^. who showed that PG545 inhibited heparanase-induced autophagy in heparanase-overexpressing (Hepa) U87 glioma cells, our results clearly identified PG545 as a promoter of autophagy in OvCa cells. We believe that these results should not be viewed as conflicting data but rather as evidence that further reinforce the concept that autophagy can act either as a promoter of cell death or a survival mechanism in a cell type-dependent fashion.

In our *in vivo* study, mice treated with a specific cPLA2 inhibitor, AACOCF3, had significantly smaller tumor burden compared to untreated controls; this suggests that a higher level of cPLA2 may play an important role in ovarian tumorigenesis. Interestingly, AACOCF3 and CBP combination therapy was more effective in delaying tumor growth compared to either drug along. It is important to note that this is the first report to show that ovarian tumor formation associated with HSulf-1 loss is regulated by higher activity of cPLA2.

## Materials and Methods

### Cell culture

The human OvCa cell lines OV202NTC, OV202Sh1, OV202Sh2 and OV202Cl7 were grown in minimum essential medium alpha 1X (Cellgro, cat# 15-012-CV) supplemented with 20% fetal bovine serum (Biowest, cat# S1620) and 1% penicillin-streptomycin. OV202 clones and NTC were grown in the presence of 1 μg/ml puromycin as a selection marker for the HSulf-1 shRNA. The growth media for the OV202Cl7 cells were additionally supplemented with 400 μg/ml of G418 as a selection marker for the HSulf-1 expression plasmid.

TOV2223 cell line was a kind gift from Dr. Anne-Marie Mes-Masson from University of Montreal, Canada.

HSulf-1^+/+^ and HSulf-1^−/−^ mouse embryonic fibroblast (MEF) cell line was a kind gift from Dr. Thomas Dierks, Bielefeld University, Bielefeld, Germany. They were grown in the DMEM 1X medium in the presence of 10% fetal bovine serum and 1% penicillin-streptomycin with addition of 650 μg/ml of G418. OV2008 cells were grown in RPMI 1640 medium (Cellgro, cat# 10-040-CV) supplemented with 10% fetal bovine serum and 1% penicillin-streptomycin.

ATG5^−/−^ MEF cell line was a kind gift from Dr. Daniel Billadeau, Mayo Clinic, Rochester^66^. These cells were grown in DMEM with 10% FBS and 4 mM L-glutamine.

### Inhibitors

AACOCF3, an inhibitor of p-cPLA2 (Cayman chemicals, cat# 62120) was used to inhibit the cPLA2 activity and LD biogenesis. MEK inhibitor U0126 (Sigma-Aldrich, cat# U120) was used to inhibit ERK activity. PG545, a heparanase inhibitor, was used to block the effect of extracellular growth factor signaling (Progen pharmaceuticals).

### Cell proliferation

Cell proliferation was quantified by MTT assay (Promega, cat# G4000) as previously described^67^.

### Generation of cPLA2 downregulated stable clone

OV202Sh1 cell line and OV2008 cells were transfected with Sh cPLA2 (ShCPLA2 1: TGATACAAATGTAGGGATATA, and ShCPLA2 2: CCTTGTATTCTCACCCTGATT) using standard transfection protocol and reagents. Stable clones were further selected by puromycin. OV202 cells were cultured in 5% CO2 - 95% air humidified atmosphere at 37 °C with minimal essential medium supplemented with 20% fetal bovine serum with their specific antibiotic selection. All cell lines were tested using a PCR-based assay and found to be free of Mycoplasma contamination.

### Western blot analysis

Western blot analysis was performed as described previously^8^. Whole cell lysates were analyzed with the following antibodies: HSulf-1 (Abcam, cat# ab31960), p-cPLA2 (Sigma, cat# SAB4503812), t-cPLA2 (SantaCruz, cat# SC454), p-ERK (Cell Signaling, cat# 9106), t-ERK (Cell Signaling, cat# 9102), LC3B (Cell Signaling, cat# 3868), GAPDH (Cell Signaling, cat# 3683), β-actin (Gene Tex, cat# GTX629630) and β-tubulin (GeneTex, cat# GTX109639).

### Detection of autophagic vesicles by Cyto-ID staining

OV202NTC and OV202Sh1 cells were seeded in the 4-well tissue culture chambered slides and treated with indicated concentrations of AACOCF3, PG545 or starved with EBSS in the presence or absence of bafilomycin. After treatment, media was removed and washed with 1xPBS and AVs were detected with a Cyto-ID autophagy detection kit (Enzo Life Sciences, cat# ENZ-51031). Images were captured using a Zeiss LSM 510 microscope.

### Detection of acidic vesicular organelles using Acridine orange staining

OV202NTC and OV202Sh1 cells were seeded in 4-well tissue culture chambered slides and grown overnight. Cells were stained the next day with acridine orange (1 μg/ml; Molecular Probes, cat# A1301) in a serum free medium for 15 minutes at 37 °C. Acridine orange was removed and images were captured in Zeiss LSM microscope. Red fluorescence indicates the acidic vesicles as described earlier^68^.

### Transmission Electron microscopy

Cultured cells were washed twice with PBS and then fixed in Trumps fixative containing 4% formaldehyde and 1% glutaraldehyde in a phosphate buffer pH ~7.3, post-fixed in 1.0% OsO4, dehydrated with ethanol gradation, and transitioned into propylene oxide for infiltration and embedding into spurr epoxy resin. Ultrathin sections were cut onto grids, stained with uranyl acetate and lead citrate, and examined with a JEM-1400 (JEOL USA) transmission electron microscope, and digitally photographed. We assessed the cytoplasmic LDs and autophagosomes morphologically and counted their number. A total of 25 cells per sample were analyzed.

### Bodipy staining

Approximately 50,000 cells were seeded on a coverslip in a 24-well plate and were grown for 24 hours in the presence of complete growth medium. Cells were washed and fixed in 4% paraformaldehyde for ten minutes at room temperature before staining with 1 μg/ml Bodipy (493/503; Sigma, cat# 790389) in PBS for ten minutes at room temperature. Coverslips were washed with PBS and mounted in a slide with Prolong Gold Antifade Reagent (Invitrogen, cat# P36935). Bodipy stained cells were examined under Zeiss-LSM 510 fluorescence microscope.

### Immunofluorescence

Cells were grown on coverslips overnight, and then fixed with 100% methanol followed by blocking with 1% BSA in PBS. After blocking, the cells were incubated with the respective primary antibodies at room temperature for 1 hour, washed three times with 1X PBS and then incubated in dark with alexa fluor goat anti-rabbit (488 nm) or with alexa fluor rabbit anti-mouse (593 nm) in 1% BSA in PBS. Coverslips were washed three times before mounting with Prolong Gold Antifade reagent (Invitrogen). Stained samples were visualized using a Zeiss-LSM 510 fluorescence microscope.

### Arachidonic acid release assay

Arachidonic acid (AA) release was evaluated in OV202NTC, OV202Sh1, OV202Sh2, OV202Cl7 cells, and OV2008 vector and clones. In a 6-well plate 0.5 × 10^6^ cells were plated and grown overnight in complete media. Growth medium was removed the following day and cells were incubated with ^3^H-AA under serum-free medium for 24 hours. The medium was removed, cells were washed three times with 1X PBS, and complete growth medium was added with or without AACOCF3 (10μM) for another 24 hours. Aliquots of growth medium from the cells were measured for radioactivity at the end of treatment. Radioactivity is showed as counts per minute (cpm/ml).

### cPLA2 activity assay

cPLA2 activity in OV202NTC, OV202Sh1, OV202Sh2 and OV202Cl7 cells was measured using cPLA2 assay kit (Cayman Chemical, cat# 765021) following manufacturer’s instructions. Data is presented as fold-change compared to control cells. This experiment was repeated twice.

### LDH release assay

In order to determine the cytotoxicity of AACOCF3, MAFP, PG545 and U0126 we conducted LDH release assays using CytoTox-One homogenous membrane integrity assay (Promega, cat# G7890). Data is calculated as percentage increase of untreated cells. This experiment was repeated twice.

### Synergy assessment using Chou-Talalay method

Constant ratio (IC_50_ over IC_50_) studies were carried out between AACOCF3 and carboplatin (CBP) carboplatin. Synergy was quantified using the Chou-Talalay method as previously described^69^,^70^,^71^ using the CalcuSyn software (Biosoft, USA). The Combination Index (CI) indicates the level of synergism or antagonism: CI less than 0.9 indicates synergism (0.3–0.7 strong; 0.7–0.85 moderate; 0.85–0.9 slight), CI between 0.9 and 1.1 nearly additive effects, and CI more than 1.1 antagonism.

### Mouse xenograft tumor model

Approximately 5 × 10^6^ OV202Sh1 cells expressing luciferase were injected intraperitoneally to nude mice. Four days following intraperitoneal inoculation the mice were treated with 10mg/kg of AACOCF3 every 3rd day or 51 mg/kg of CBP every 5 days or combination of CBP (51 mg/kg) and AACOCF3 (10mg/kg). The treatments were continued until the end of the study (28 days). All the animals were sacrificed by the 28^th^ day of the experiment. Mayo Clinic Animal Care and Use Committee approved all procedures. All experimental use of animals will comply with the guidelines of the Animal Care and Use Committee at the Mayo Foundation, in accordance with approved protocols.

### *In vivo* bioluminescence measurements

Weekly bioluminescent reporter imaging was performed to monitor the seeding of OV202Sh1-luc cells, using the Xenogen IVIS 200 System (Caliper life sciences) as previously described^48^.

### Immunohistochemistry

Immunohistochemical staining was done on tumors from control, F3, CBP and F3 + CBP treated mice. Paraffin embedded tissue sections from the tumors were stained for Ki67 and p-cPLA2. Standard immunohistochemistry was performed at the Pathology Research Core laboratory (Mayo Clinic, Rochester, MN) as previously described^48^.

### TUNEL Staining

Terminal dUTP Nick End-Labeling (TUNEL) assay on frozen tissue sections of tumors from control, F3, CBP and F3 + CBP treated mice was performed using ApopTag Fluorescein Direct *In Situ* Apoptosis Detection Kit (Millipore, cat# S7110) according to the manufacturer’s instructions. Green fluorescence indicates TUNEL staining and red fluorescence indicates propidium iodide staining.

### Image analysis

Quantification of fluorescence intensity of the confocal images were obtained with the use of ImageJ software. Fluorescence intensity was expressed as corrected total cell fluorescence (CTCF). CTCF was determined as integrated density-(area of selected cell x mean fluorescence of background reading) and expressed as an average in 25 cells (N = 2)^72^.

### Statistical analyses

All results were expressed as mean ± standard deviation. Data obtained from three separate experiments. All statistical analyses were performed using the Graphpad Prism software (San Diego, CA). Data were analyzed using t test. A level of *p* less than 0.05 was considered statistically significant, unless otherwise noted.

## Additional Information

**How to cite this article**: Roy, D. *et al*. Loss of HSulf-1: The Missing Link between Autophagy and Lipid Droplets in Ovarian Cancer. *Sci. Rep.* **7**, 41977; doi: 10.1038/srep41977 (2017).

**Publisher's note:** Springer Nature remains neutral with regard to jurisdictional claims in published maps and institutional affiliations.

## Supplementary Material

Supplementary Data
