# Supplementary material for: Loss of HSulf-1: The Missing Link between Autophagy and Lipid Droplets in Ovarian Cancer
Source: Sci Rep. 2017 Feb 7;7:41977. doi: 10.1038/srep41977 (PMC5294412; doi:10.1038/srep41977)
Supplement: Supplementary Data [file srep41977-s1.pdf]

Loss of HSulf-1: The Missing Link between Autophagy and Lipid Droplets in Ovarian Cancer

Debarshi Roy[1], Susmita Mondal[1], Ashwani Khurana[1], Deok-Beom Jung[1], Robert Hoffmann[1], Xiaoping He[1], Eleftheria Kalogera[2], Thomas Dierks[3], Edward Hammond[4], Keith Dredge[4] and Viji Shridhar[1]

[1]*Department of Experimental Pathology, Mayo Clinic, Rochester, MN, USA*
[2]*Division of Gynecologic Surgery, Mayo Clinic, Rochester, MN, USA*
[3]*Department of Chemistry, Biochemistry I, Bielefeld University, Bielefeld, Germany*
[4]*Zucero Therapeutics, Brisbane, Queensland, Australia*

Address and Correspondence
Dr. Viji Shridhar
Department of Experimental Pathology
Mayo Clinic, Rochester, MN 55905
Tel # 507-266-2775
Email: shridhar.vijayalakshmi@mayo.edu

Figure S1

A OV202 NTC

B OV202 Sh1

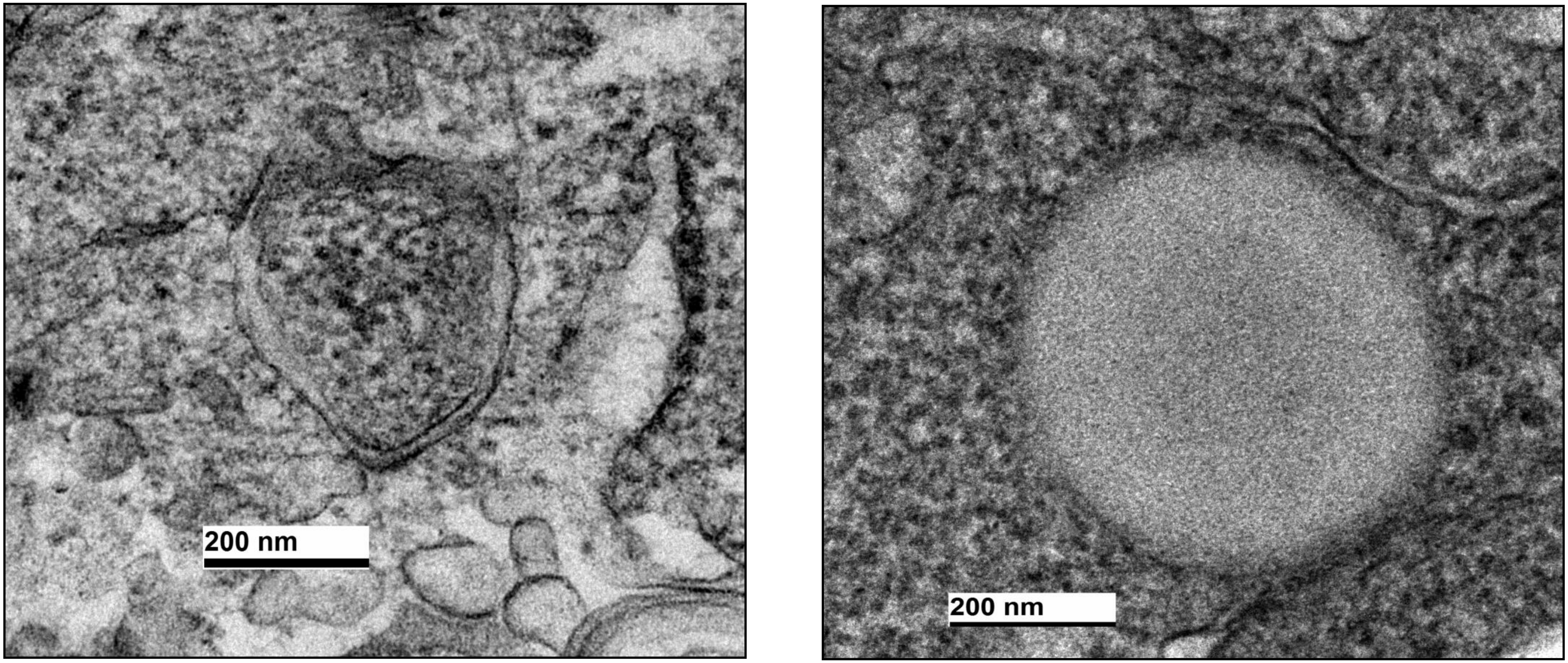

C

OV202 Sh2

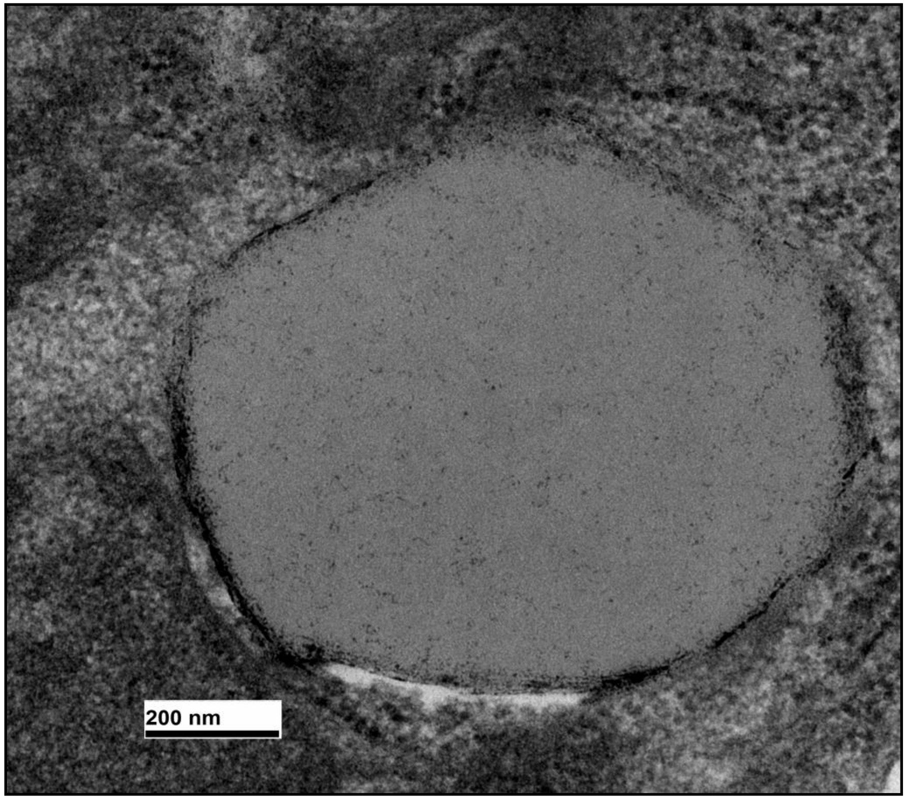

D

OV202 Cl7

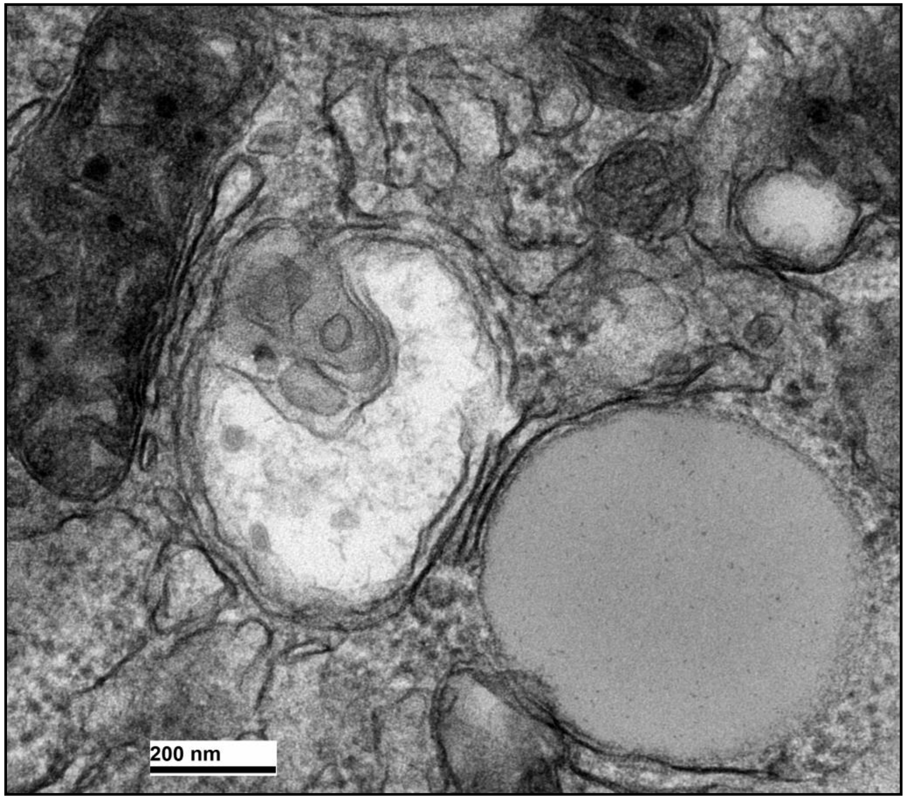

Fig S1

EM micrographs of OV202 NTC, Sh1, Sh2 and Cl7 cells. The images demonstrated here taken in a very high zoom to visualize autophagic structures and lipid droplets..

Figure S2

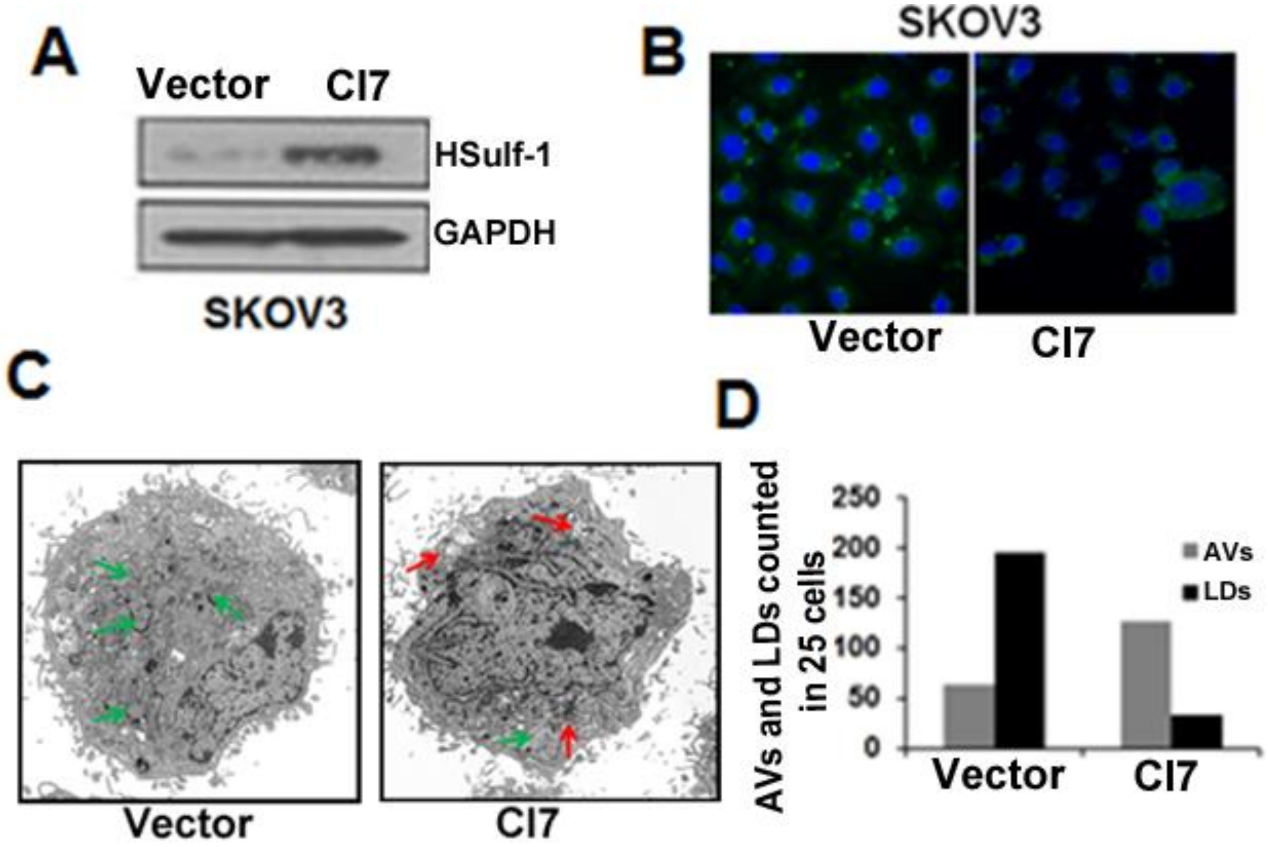

Fig S2

A. Western blot analysis of HSulf-1 expression in SKVO3 vector and Cl7 cells with GAPDH as loading controls. **B.** LD levels determined by Bodipy staining (green) in SKOV3 vector and Cl7 with nuclei stained blue with DAPI. **C.** Representative transmission electron micrograph images (TEM) of SKOV3 vector and Cl7 cells are shown; LDs and AVs are indicated by green and red arrows respectively. **D.** Quantification of LDs and AVs from form 25 cells in figure S2D is shown as bar diagram.

Figure S3

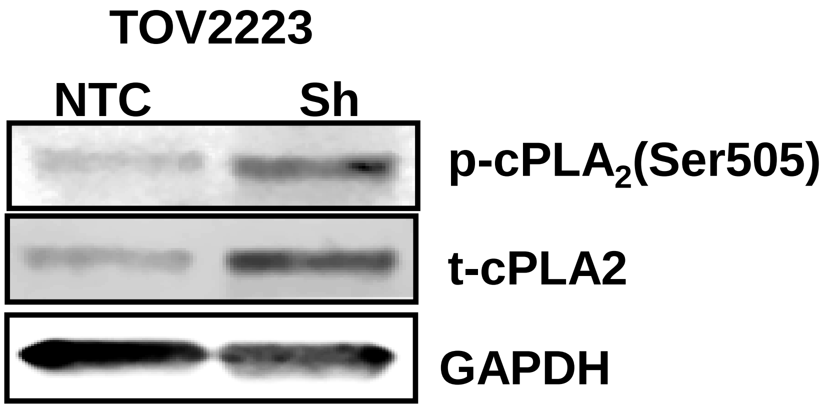

Fig S3
Protein expression of p-cPLA2 (ser505) and t-cPLA2 was determined by western blot in TOV2223 NTC and Sh cells with GAPDH as loading control.

Figure S4

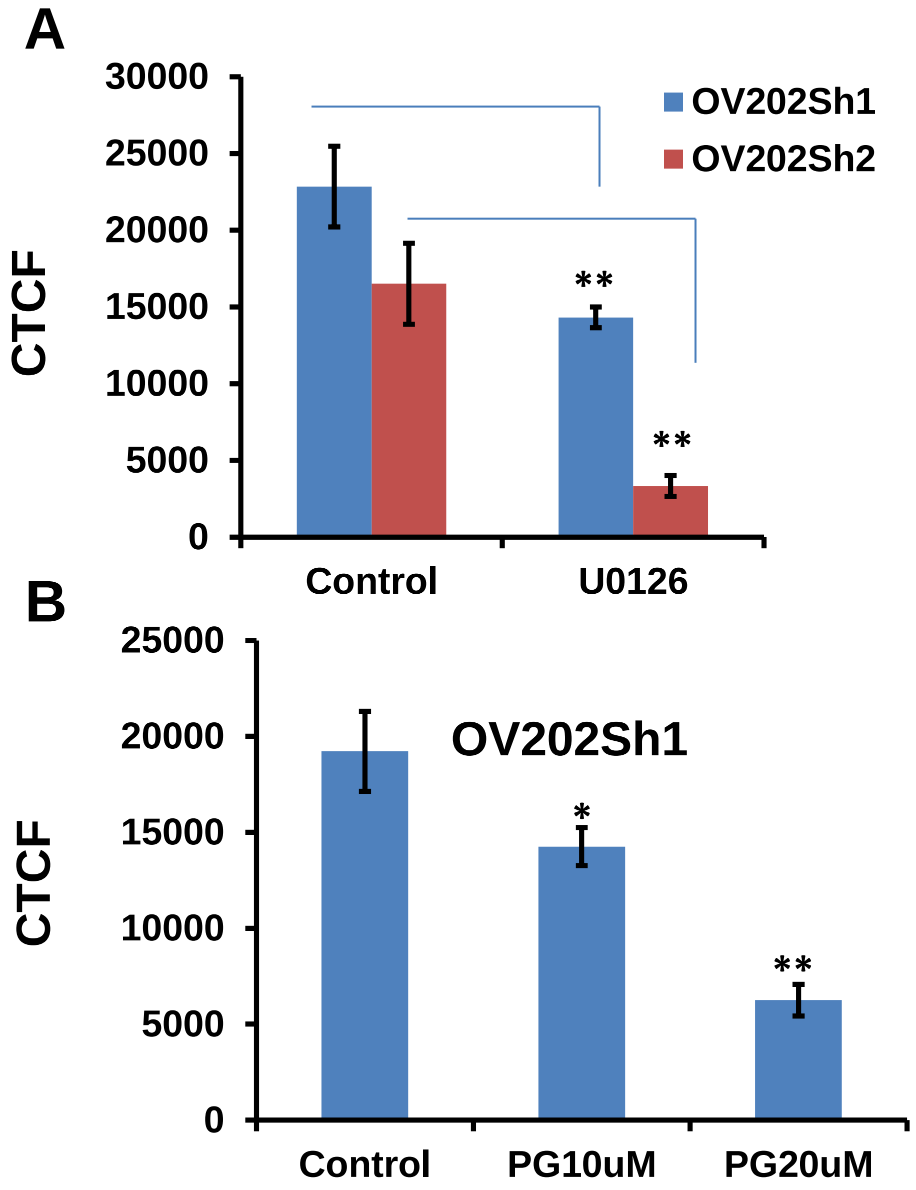

Fig S4

A. Fluorescence intensity of Bodipy is determined in OV202Sh1 and Sh2 cells after U0126 treatment. **B.** Fluorescence intensity of Bodipy Intensity is measured in OV202 Sh1 cells after treatment with PG545. Intensity of Bodipy staining in 25 cells is measured in each case using ImageJ software and represented as corrected total cell fluorescence (CTCF). *P < 0.05; **P < 0.01

Figure S5

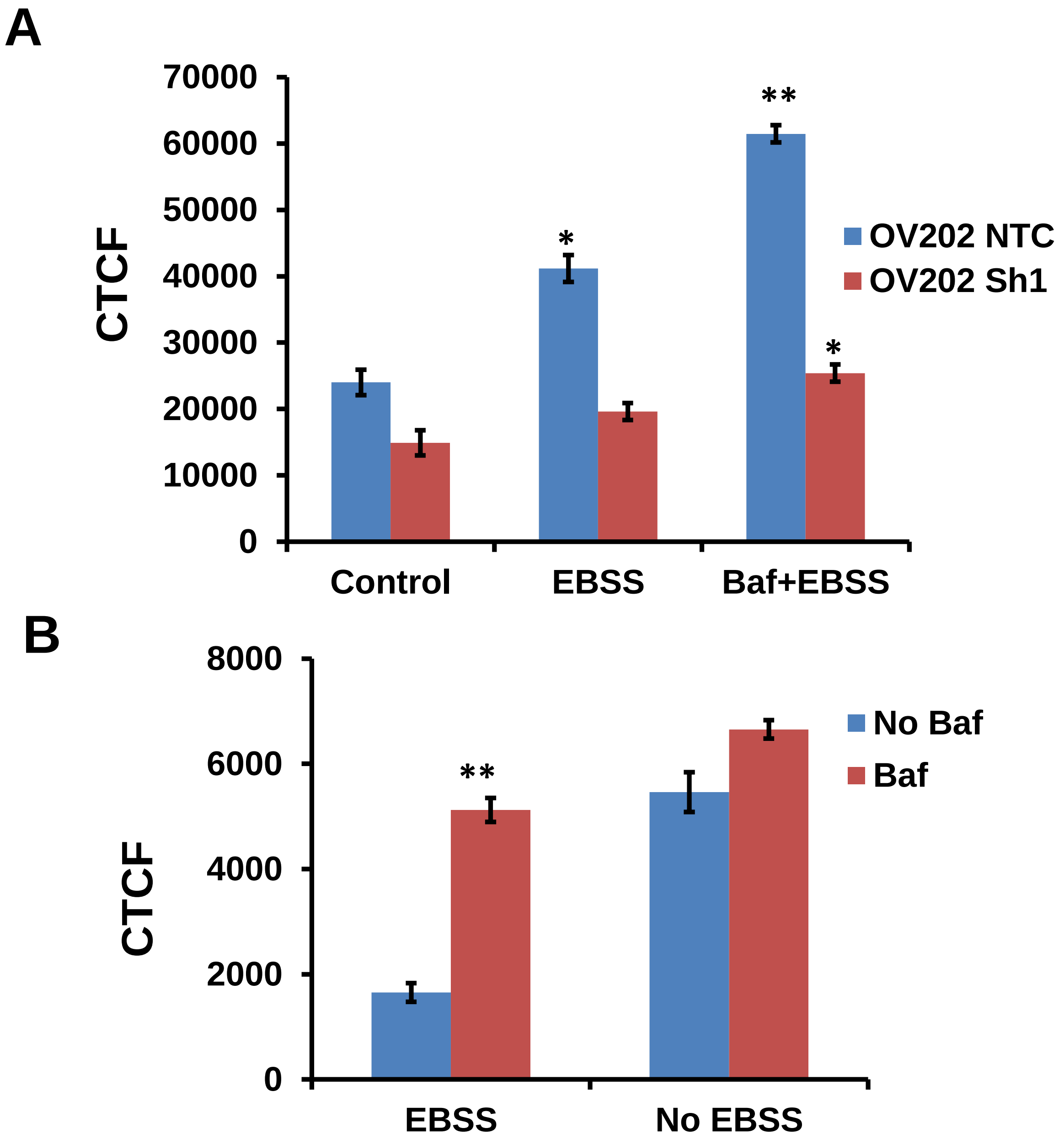

Fig S5

A. Fluorescence intensity of Cyto-ID and Bodipy intensity (**B**) is determined in OV202NTC and Sh1 cells after EBSS treatment with and without bafilomycin. Intensity of Cyto-ID and Bodipy in 25 cells is measured in each case using ImageJ software and represented as corrected total cell fluorescence (CTCF). *$P < 0.05$; **$P < 0.01$

Figure S6

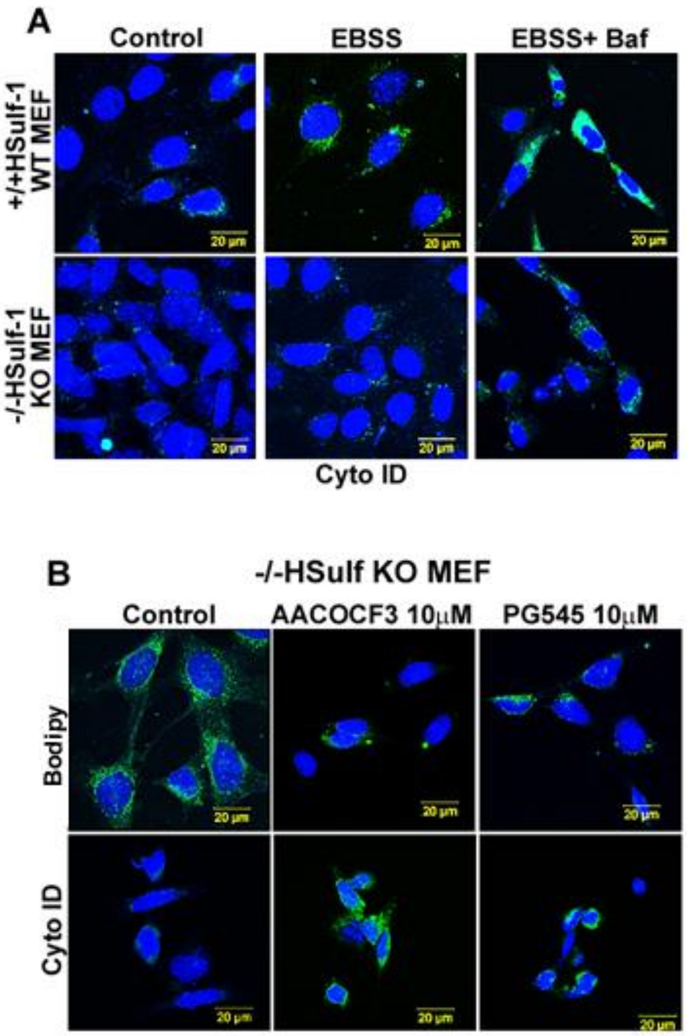

Fig S6

A. Mouse Embryonic Fibroblast (MEF) wild type (WT) and HSulf-1 knock out (Sulf-1 $^{-/-}$) cells were either grown in complete media (CM) or starved with EBSS in the presence and absence of bafilomycin A1 followed by Cyto-ID staining to demonstrate the autophagic vesicles. **B.** MEFs were treated with 10μm of AACOCF3 and PG545 respectively, and then stained with either Bodipy or Cyto-ID to detect LDs and AVs.

Figure S7

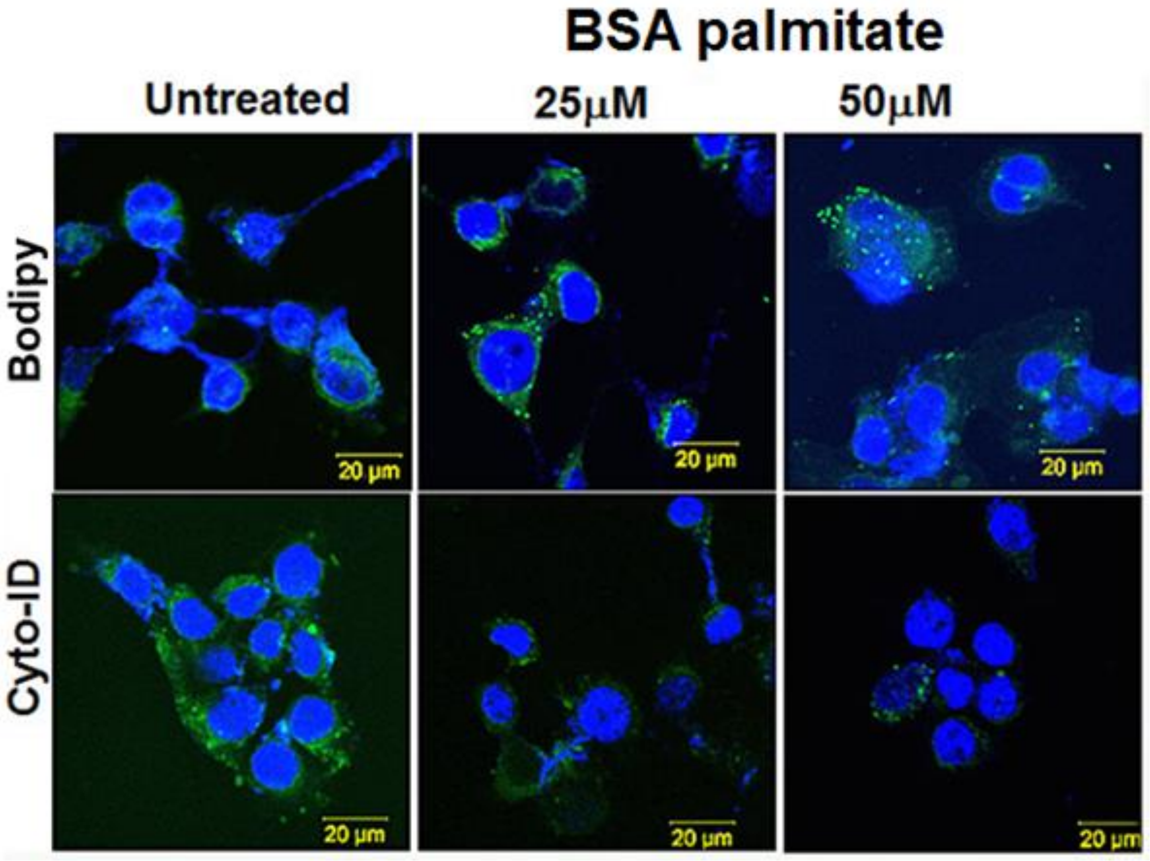

Fig S7
OV202NTC cells were treated with 25 and 50µM of BSA conjugated sodium palmitate for 24 hrs. After treatment, cells were labeled with Bodipy and Cyto-ID to detect lipid droplets and autophagic vesicles respectively.

Figure S8

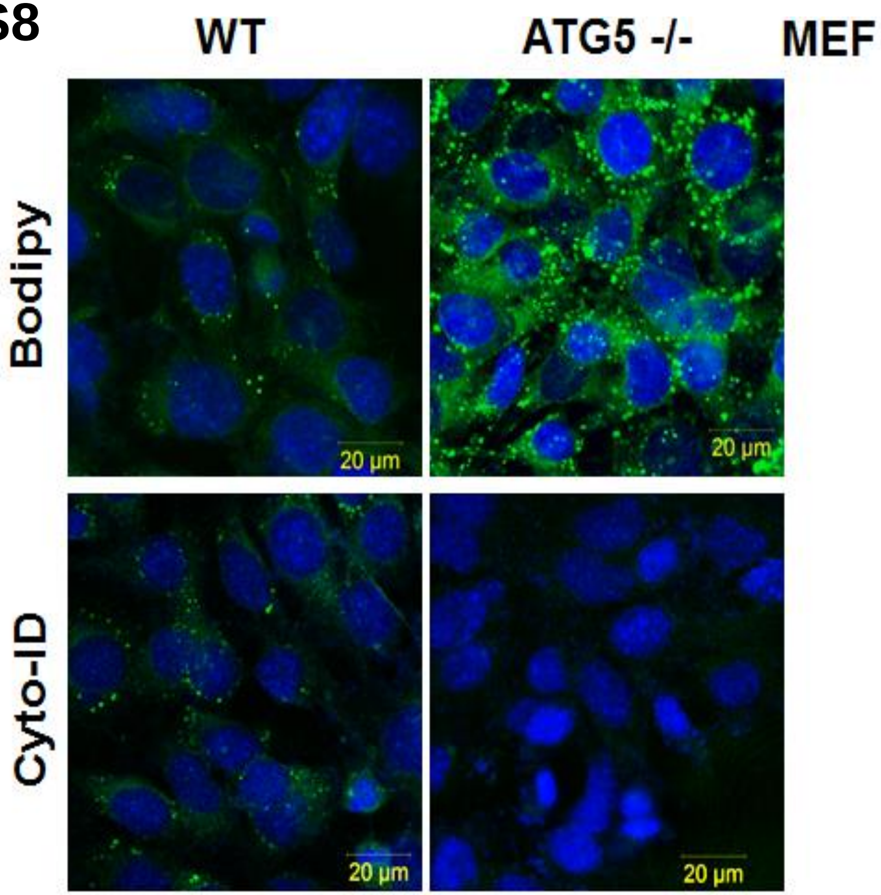

Fig S8
Wild type (WT) and ATG 5 knockdown mouse embryonic fibroblast (MEF) cells were stained with Bodipy and/or Cyto-ID to identify lipid droplets and autophagic vesicles.

Figure S9

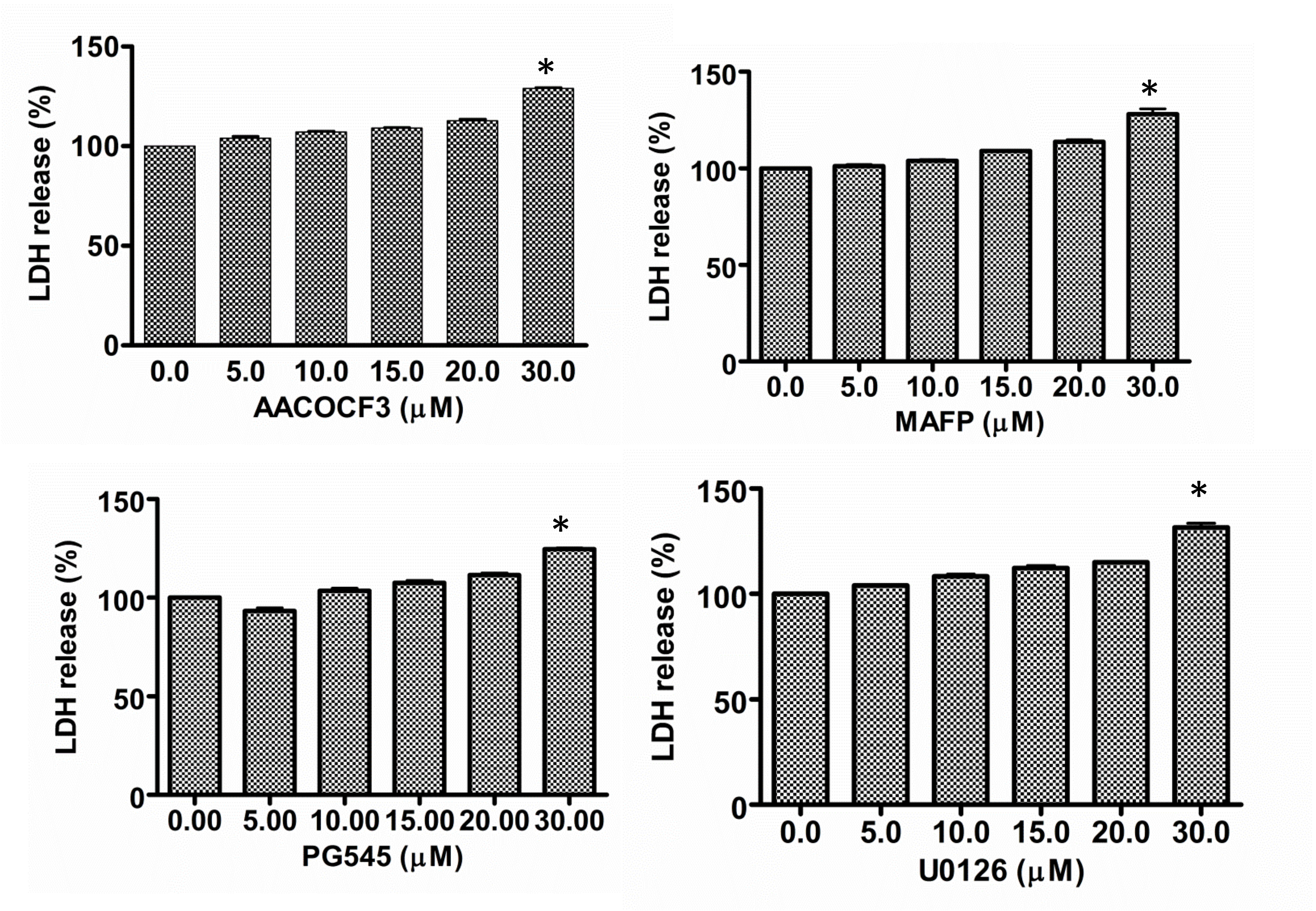

Fig S9
LDH release assay in OV202Sh1 cells treated with different concentrations of AACOCF3, PG545, MAFP and U0126 for 24 hours.
The data is calculated as the percentage increase of untreated cells.

Figure S10

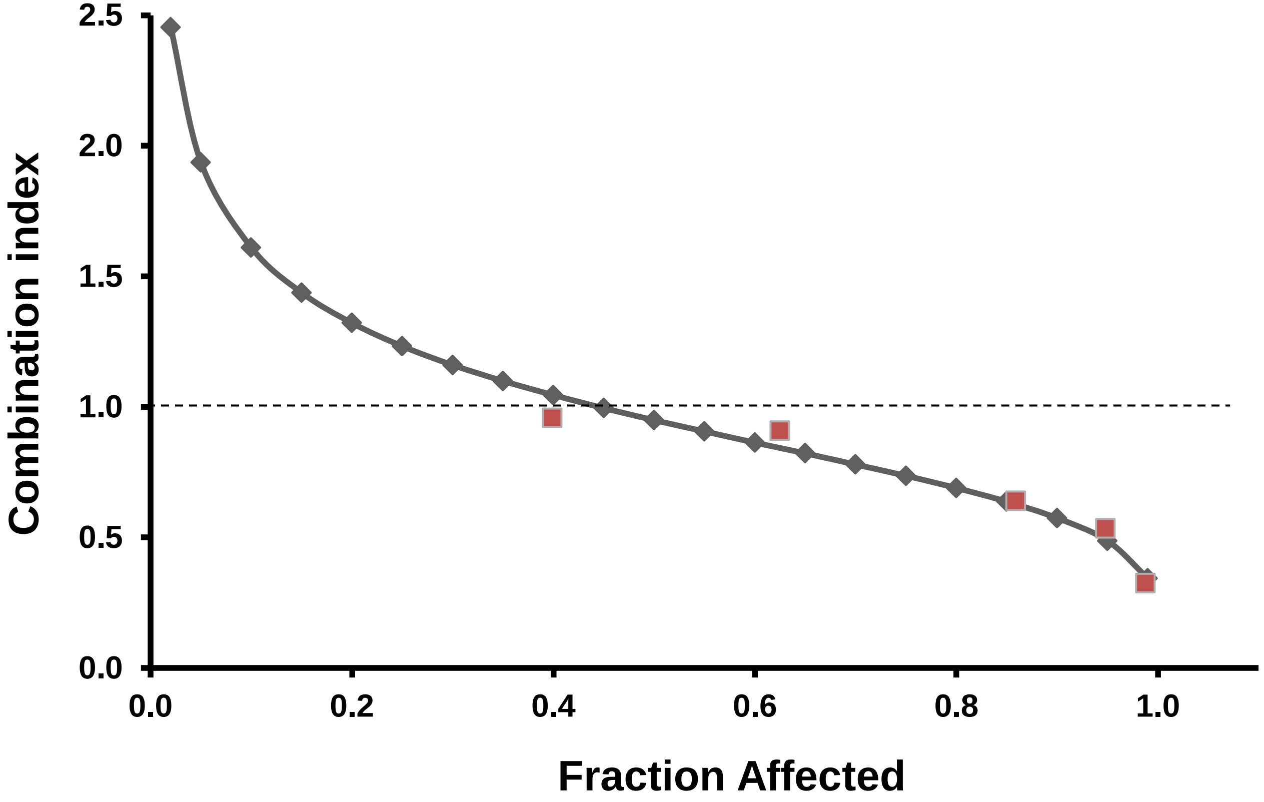

Fig S10
Fractional effect-Combination index plots of CBP and F3 combination treatment. The effect of the combination treatment was analyzed using CalcuSyn software in OV202 Sh1 cells.
